# Partial Discharge Monitoring on Metal-Enclosed Switchgear with Distributed Non-Contact Sensors

**DOI:** 10.3390/s18020551

**Published:** 2018-02-11

**Authors:** Chongxing Zhang, Ming Dong, Ming Ren, Wenguang Huang, Jierui Zhou, Xuze Gao, Ricardo Albarracín

**Affiliations:** 1 State Key Laboratory of Electrical Insulation for Power Equipment, Xi’an Jiaotong University, Xi’an 710049, China; zhangcx.123@stu.xjtu.edu.cn (C.Z.); dongming@mail.xjtu.edu.cn (M.D.); wghuang@stu.xjtu.edu.cn (W.H.); ronaldo@stu.xjtu.edu.cn (J.Z.); slg1114@stu.xjtu.edu.cn (X.G.); 2 Electrical and Electronic Engineering, Automatic Control, and Applied Physics, Escuela Técnica Superior de Ingeniería y Diseño Industrial, Universidad Politécnica de Madrid, Ronda de Valencia 3, 28012 Madrid, Spain

**Keywords:** partial discharge (PD), transient earth voltage (TEV), wireless sensor network (WSN), insulation diagnosis

## Abstract

Metal-enclosed switchgear, which are widely used in the distribution of electrical energy, play an important role in power distribution networks. Their safe operation is directly related to the reliability of power system as well as the power quality on the consumer side. Partial discharge detection is an effective way to identify potential faults and can be utilized for insulation diagnosis of metal-enclosed switchgear. The transient earth voltage method, an effective non-intrusive method, has substantial engineering application value for estimating the insulation condition of switchgear. However, the practical application effectiveness of TEV detection is not satisfactory because of the lack of a TEV detection application method, i.e., a method with sufficient technical cognition and analysis. This paper proposes an innovative online PD detection system and a corresponding application strategy based on an intelligent feedback distributed TEV wireless sensor network, consisting of sensing, communication, and diagnosis layers. In the proposed system, the TEV signal or status data are wirelessly transmitted to the terminal following low-energy signal preprocessing and acquisition by TEV sensors. Then, a central server analyzes the correlation of the uploaded data and gives a fault warning level according to the quantity, trend, parallel analysis, and phase resolved partial discharge pattern recognition. In this way, a TEV detection system and strategy with distributed acquisition, unitized fault warning, and centralized diagnosis is realized. The proposed system has positive significance for reducing the fault rate of medium voltage switchgear and improving its operation and maintenance level.

## 1. Introduction

With 10 kV and 35 kV metal-enclosed switchgear being widely used in power distribution networks, their safe operation is directly related to the reliability of the power system, as well as the power quality on the consumer side [1,2,3]. According to statistics, nearly 40% of the faults in switchgear are originated from insulation faults or potential defects such as cracks in the insulator [4], bad electrical contacts, and pollution of the insulating bush. These insulation defects can excite partial discharge (PD) under electric fields that are hazardous to insulation but can also be detected for insulation diagnosis. To realize online detection, methods such as ultra-high-frequency (UHF) method [5,6,7,8], acoustical emission (AE) method [9,10,11], and transient earth voltage (TEV) method [12,13,14] have been proposed to couple PD signals based on the phenomena of electromagnetic (EM) radiation, acoustic radiation, and transient current flow that accompany PDs, respectively [15]. Among these methods for online detection of discharge-type insulation failure, the TEV method is the most acceptable for PD detection of metal-enclosed switchgear because of its high sensitivity, broad frequency band, easy installation, and non-electrical contact detection [16,17,18]. However, over the years, PD monitoring and insulation status evaluation have been implemented manually using professional PD instrumentation with scheduled testing periods [19]. This application mode is not sufficient to meet the demands of real-time monitoring, low labor cost, and the growing number of metal-enclosed switchgear in developing power systems. Furthermore, the subjectivity of manual operation may result in increased errors (missing and false judgment) in PD detection in the long term. Therefore, although there is a certain prevention and assessment capability with current TEV technology for switchgear insulation failure, its application effect is unsatisfactory. The various problems that currently exist are not due to limitations of the TEV detection technology itself, but a lack of a suitable TEV detection application method with sufficient technical cognition and analysis. Thus, to better apply TEV detection technology to the fault warning of switchgear equipment, it is necessary to further innovate its application mode and exploit its technical potential.

This paper proposes an innovative online PD detection system and corresponding application strategy based on an intelligent feedback distributed TEV wireless sensor network (WSN), consisting of a central server and TEV sensing nodes distributed on each switchgear. The principle of TEV generation and detection are introduced in Section 2. In Section 3.1, the realization principle of wireless TEV detection system, which mainly consists of sensing, communication, and diagnosis layers is established. Then, the TEV coupling module and preprocessing circuit are designed and experimentally evaluated in Section 3.2 and Section 3.3, respectively. In Section 3.4 and Section 3.5, the wireless TEV sensor is manufactured and calibrated. In addition, some essential parameters or indices are also introduced and explained. In Section 4, it is established the topological structure of WSN and the detailed application strategy of the wireless TEV sensor. In Section 5, based on the designed WSN, the PD diagnostic methods of switchgear, which include elementary status diagnosis and in-depth status diagnosis, are formed and obtained. The conclusions are summarized in Section 6. Finally, a TEV detection system and strategy with distributed acquisition, unitizing fault warning, and centralized diagnosis is carried out.

## 2. Principle of TEV Detection

**Figure 1:**
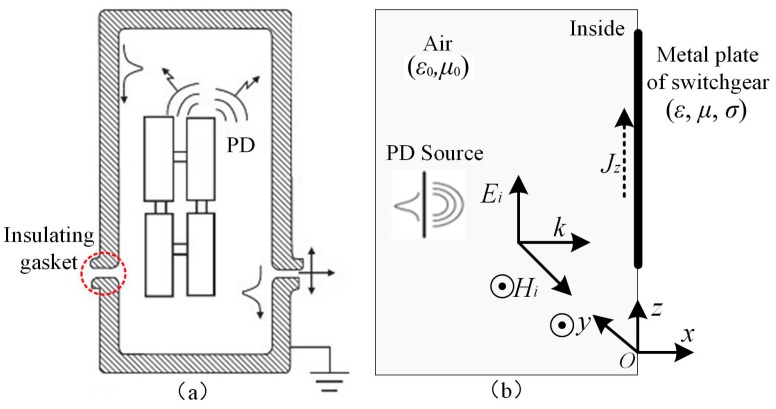
Principle of TEV signal coupling: (**a**) Propagation of PD EM waves; (**b**) generation of transient earth voltage (*ε*_0_: permittivity of air; *μ*_0_: magnetic conductivity of air; *H_i_*: magnetic field intensity of incident electromagnetic wave; *E_i_*: electric field component of incident electromagnetic wave; *ε*: permittivity of metal plate; *μ*: magnetic conductivity of metal plate; *σ*: conductivity of metal plate; *k*: direction of electromagnetic wave propagation; and *J_z_*: induced current density in conductor).

The charges excited by discharges in high-voltage (HV) installations accumulate at the metal linkage of ground point initially and quickly forms a current that flows to earth and spreads on the surface of the apparatus. Several discontinuous shielding points exist in a switchgear, such as the insulator, insulated pillar, and flange junction, where high-frequency PD signals can leak from the shielding enclosure [20] and propagate onto the outer surface of the metal, as shown in Figure 1. This series of transient impulse signals is called TEV [21]. The coupled voltage signal that passes on the surface of the metal can be obtained by a capacitive sensor. The structures of actual switchgear and the switchgear model with artificial defects used in tests are not the complete EM shielding type; therefore, the high-frequency EM waves spread out from the metal chamber and form transient earth voltage on the surface of the metal.

In order to simplify the analysis, a plane electromagnetic wave that propagates in the direction of the *x*-axis in air and radiates out from the insulating gasket of metal-enclosed switchgear, can be considered. For the wave, its electric field *E_i_* and magnetic field *H_i_* can be expressed by the following equations [21]:

(1) $$E_{i}=e_{z}E_{z}=e_{z}\varepsilon_{0}e^{-j\beta x}$$

(2) $$H_{i}=-e_{y}H_{y}=-e_{y}\frac{\varepsilon_{0}}{\eta_{0}}e^{-j\beta x}$$

(3) $$\beta =\omega \sqrt{\mu_{0}\varepsilon_{0}},\;\eta_{0}=\omega \sqrt{\frac{\mu_{0}}{\varepsilon_{0}}},\;\gamma =\frac{1+j}{\delta },\;\delta =\sqrt{\frac{1}{\pi f\mu \sigma }}$$

where *η*_0_ is the characteristic impedance of air; *β* is the phase constant of EM wave; *δ* is the skin depth of the EM wave in the conductor (metal surface of switchgear); *γ* represents the propagation constant; *e_y_* and *e_z_* represent the unit vector in the *y* and *z* directions, respectively; ω is the angular frequency of the electromagnetic wave; and *f* is the frequency of the electromagnetic wave.

The wave equation satisfied by the electric field *E_z_* is derived as follows [22]:

(4) $$\frac{d^{2}E_{z}}{dx^{2}}-\gamma^{2}E_{z}=0$$

It is assumed that the electric field intensity on the plane *x* = 0 is expressed by the following equation.

(5) $$E=e_{z}E_{0}$$

where *E*_0_ represents the parallel electric field at the surface of the metal plate. According to Equations (4) and (5), the electric field in the conductor can be obtained as follows:

(6) $$E_{z}=E_{0}e^{-x/\delta }e^{-jx/\delta }$$

Then, the induced current density in the conductor can be obtained as follows:

(7) $$J_{z}=\sigma E_{z}=\sigma E_{0}e^{-x/\delta }e^{-jx/\delta }$$

The total current on the metal plate per unit area can be expressed by the following equation:

(8) $$I_{z}=\iint J_{z}dS=\frac{\sigma \delta E_{0}}{1+j}$$

When the metal shell of the switchgear induces the charge (*Q*) because of the surface current, a capacitive sensor (*C*_1_) can couple the charge and output a transient earth voltage signal (*U*), thus:

(9) $$\frac{dU}{dt}=\frac{dQ}{C_{1}dt}=\frac{1}{C_{1}}\iint_{S}I_{z}dS$$

According to Equations (8) and (9), the amplitude of the TEV signal can be obtained as shown by the following equation:

(10) $$U=\frac{\sigma \delta }{C_{1}(1+j)}\int \left(\iint_{S}E_{0}dS\right)dt$$

Therefore, the transient voltage signal on the surface of the switchgear can be coupled and measured by a capacitive sensor.

## 3. Hardware Design of Wireless TEV Sensors

### 3.1. Design Principle of the Wireless TEV Detection System

**Figure 2:**
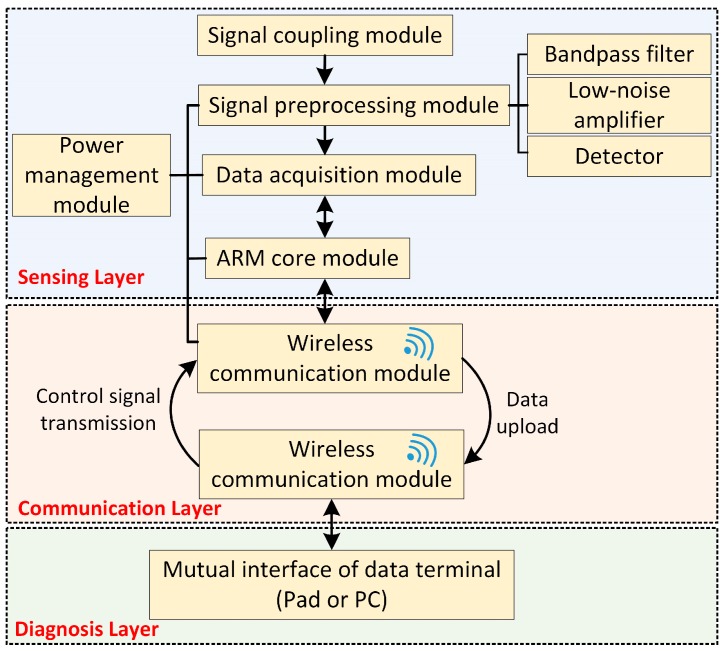
Overall design structure of the wireless TEV detection system.

The wireless TEV detection system consists of respective sensing, communication, and diagnosis layers, as shown in Figure 2. The sensing layer is composed of the WSN. Every sensing unit consists of several basic modules—specifically, a TEV signal coupling module, signal preprocessing module, data acquisition module, wireless communication module, power management module, and ARM core. The selected ARM core module in this study is the STM32f205_64pin (STMicroelectronics, Geneva, Switzerland) with the basic frequency of 120 MHz and 64K RAM. The wireless TEV sensors are used to detect the PD signal and operation state of the switchgear. The communication layer is connected to the sensing layer and the diagnosis layer. The distributed sensors upload the monitoring data to the diagnosis layer regularly; the diagnosis layer transmits the control command or monitoring strategy to the distributed sensors. In addition, the sensing layer only uploads abnormal warning messages to the diagnosis layer after detecting abnormalities, which avoids reporting redundant data and consumes less power. There are two main functions regarding the diagnosis layer. First of all, the uploaded abnormal data is analyzed and diagnosed. In addition, this layer can also determine a dynamic monitoring strategy to automatically adjust the time interval of monitoring according to the current operation and load status of switchgear.

### 3.2. TEV Coupling Module

**Figure 3:**
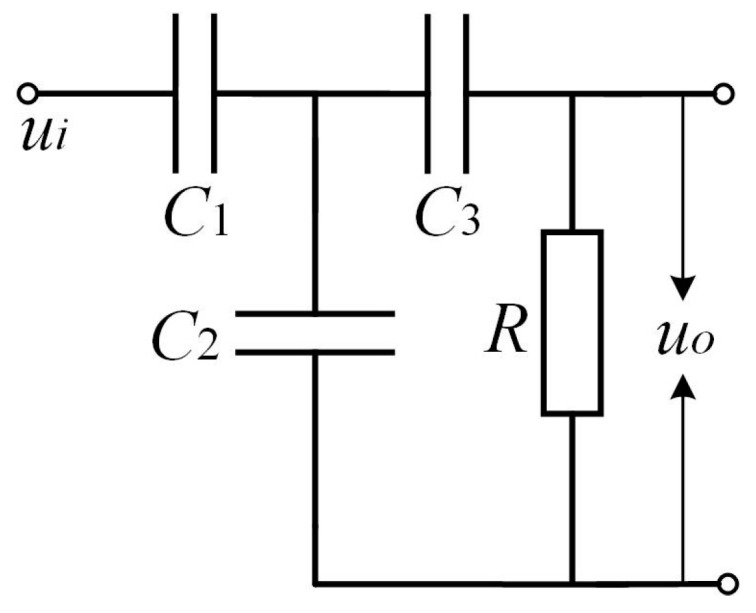
Circuit schematic of the TEV sensing unit.

A schematic circuit of the TEV coupling module is shown in Figure 3. In the schematic, *C*_1_ is the equivalent capacitance between the metal shell of the switchgear and the TEV sensor, *C*_2_ is the filter capacitor, *C*_3_ is the coupling capacitance, *R* is the resistance, *u_i_* represents the induced transient voltage on the switchgear surface and *u_o_* is the output voltage of TEV coupling module.

On the basis of loop analysis, the circuit transfer function is expressed as follows:

(11) $$T(s)=\frac{u_{o}(s)}{u_{i}(s)}=\frac{1}{1+\frac{C_{2}}{C_{1}}+\frac{\left(C_{1}+C_{2}+C_{3}\right)}{C_{1}C_{3}R\times s}}$$

In order to optimize the detection sensitivity, the denominator of the transfer function should be as large as possible. When *C*_1_ is much greater than *C*_2_ and *C*_3_ = *kC*_1_, the transfer function can be simplified to the following equation:

(12) $$T(s)=\frac{u_{o}(s)}{u_{i}(s)}=\frac{1}{1+\frac{1+k}{kC_{1}R\times s}}$$

To satisfy the high sensitivity requirement:

(13) $$\frac{\left(1+k\right)}{wkC_{1}R}<<1$$

here, *w* represents the angular frequency and *w* = 2π*f*.

Then, the value of *R*_1_ can be derived:

(14) $$R>>(1+\frac{1}{k})\frac{1}{wC_{1}}$$

**Figure 4:**
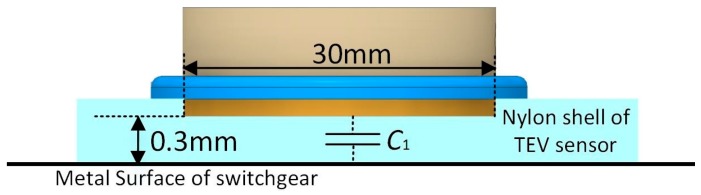
Determination of capacitance *C*_1_.

The specific process of parameter determination is as follows. Firstly, the value of *C*_2_ and *C*_3_ are set to 1 pF and 100 pF, respectively. Then, as it is previously mentioned, the value of capacitance *C*_1_, which is the equivalent geometric capacitance between the metal surface of the switchgear and the TEV coupling module, should be as bigger as possible than *C*_2_. The shell of TEV sensor is made from nylon, whose relative dielectric constant is 4.1 and the thickness of the dielectric layer is 0.3 mm. In addition, considering the volume of the sensor, the diameter of the circular metal surface is 30 mm, as shown in Figure 4.

The value of coupling capacitor *C*_1_ can be calculated according to the following equation:

(15) $$C_{1}=\frac{\varepsilon S}{d}=\frac{\varepsilon_{0}\varepsilon_{r}\pi r^{2}}{d}=\frac{4.1\times 8.85\times 10^{-12}\times \pi \times 0.015^{2}}{3\times 10^{-4}}\approx 85\;\mathrm{pF}$$

(16) $$\mathrm{Coefficient}\;k=\frac{C_{3}}{C_{1}}=\frac{100\;\mathrm{pF}}{85\;\mathrm{pF}}\approx 1.18$$

The main frequency-domain distribution of TEV signals are far less than 100 MHz (*f*_max_ = 10^8^ Hz), so the value of resistance *R* could be determined:

(17) $$R>>(1+\frac{1}{k})\frac{1}{wC_{1}}=(1+\frac{1}{1.18})\times \frac{1}{2\times 3.14\times 10^{8}\times 85\times 10^{-12}}=34.6\;\Omega$$

Thus, the value of *R* is determined to be 5 kΩ and the determined value of each component is as shown in Table 1.

**Figure 5:**
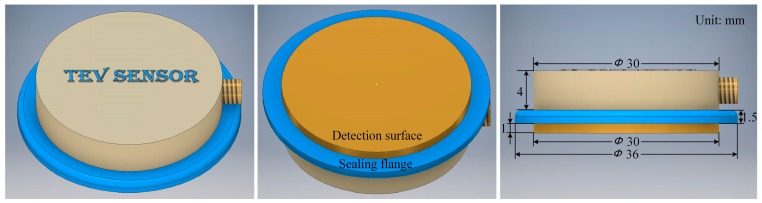
TEV coupling module.

According to the optimized component parameters, the TEV coupling module is designed and manufactured, as shown in Figure 5. The circular detection surface, which makes up the capacitor *C*_1_ with the dielectric layer and switchgear surface (Figure 4), is made from brass and its diameter is 30 mm. The extra sealing flange is used for the convenience of assembly. The TEV coupling module outputs the PD signal by a SMA coaxial connector.

**Figure 6:**
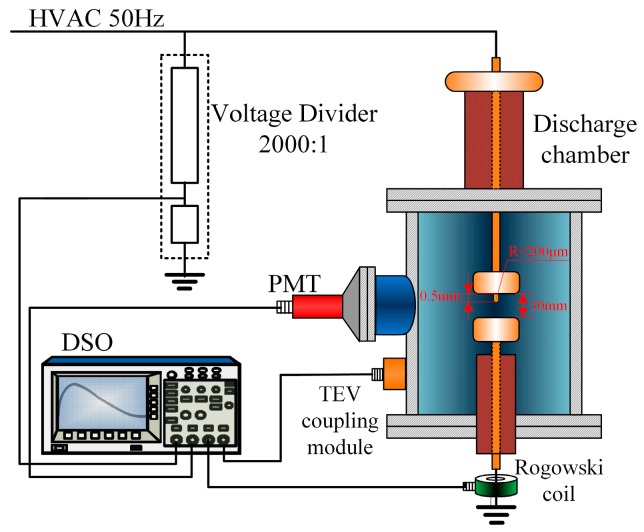
Schematic diagram of the PD measurement system.

In order to verify the performance of the TEV coupling module, a PD detection experimental switchgear system with metal protrusion defect was devised, as shown in Figure 6. A stabilized power frequency HV supply was connected to the plate electrode via a water resistor (500 kΩ). The designed TEV module was attached on the surface of discharge chamber to couple the PD signal. In addition, the experimental system also introduced a photomultiplier tube (PMT, HAMAMATSU, R7600U-01, Shizuoka, Japan) for the photon radiation intensity detection and Rogowski coil for the pulse current signal in the discharge process. Finally, signals from voltage divider (2000:1), a Rogowski coil and PMT were fed to a digital oscilloscope (LECROY, 64MXs-B, New York, NY, USA) with an analogue bandwidth of 600 MHz and 10 Gs/s sampling rate.

**Figure 7:**
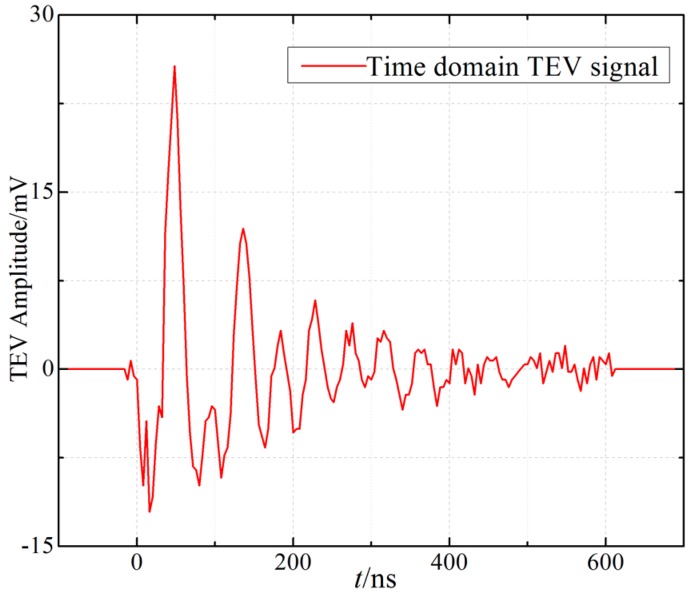
Time-resolved PD pulse waveform based on the designed TEV coupling module.

**Figure 8:**
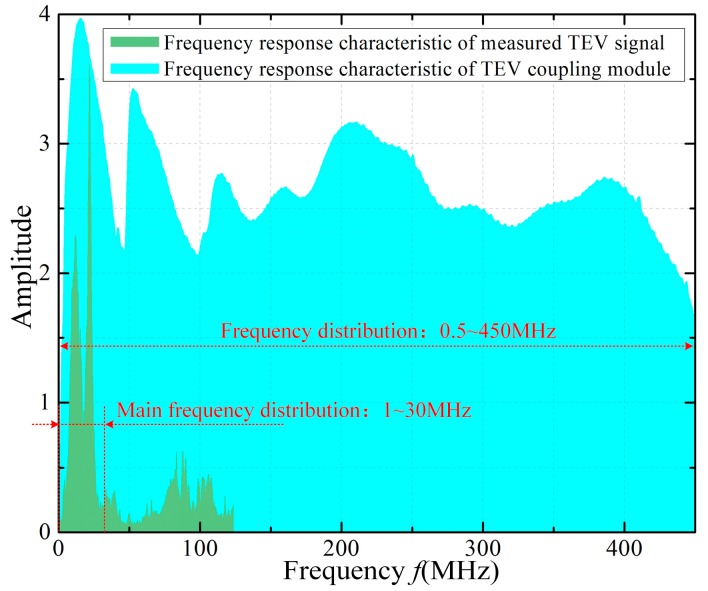
Frequency response of the measured TEV signal and TEV coupling module.

Figure 7 shows the measured TEV signal under the applied voltage of 11.5 kV in air, which has the feature of oscillating attenuation. The duration of the TEV signal is approximately 0.6 µs and, according to Figure 8, its main frequency spectrum ranges from 1 MHz to nearly 30 MHz, which is also set as the upper cut-off frequency of the filter circuit in the ensuing sections. Using a spectrum analyzer (GWINSTEK, GSP-930, New Taipei City, Taiwan) with 9 kHz–3 GHz bandwidth and the frequency resolution of 1 Hz, the frequency response of the TEV coupling module was obtained (see Figure 8). The spectrum distribution range is almost up to 450 MHz, which contains the frequency components of the PD signal.

### 3.3. Preprocessing Circuit Wireless TEV Sensor

The frequency of the TEV intrinsic signal is as high as dozens of MHz (see Figure 8). Hence, the conventional TEV PD detector has an acquisition unit with a high-speed sampling rate. However, the online monitoring distributed TEV sensing unit needs to be low-cost, low-power, and compact; thus, down-detected sampling has to be carried out instead of high-speed sampling. Actually, the envelope of the TEV signal, which contains the fault characteristics and phase information (reference phase, explained in Section 5.3.1) of the partial discharge, can be used for insulation diagnosis of switchgear.

**Figure 9:**
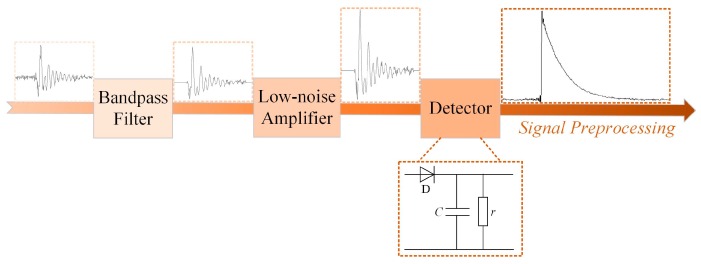
TEV Signal preprocessing procedure.

The TEV signal preprocessing procedure is shown in Figure 9 and its corresponding circuit consists of a bandpass filter, low-noise amplifier, and detector. The original high-frequency signal coupled by TEV coupling module is filtered and amplified, then it is converted into low-frequency double-exponential signal via the designed detector, which includes three main components: diode (D), capacitor (*C*), and resistor (*r*). The technical principle of detector is that when the amplitude of signal is larger than the forward voltage of diode D, the diode D is turned on and its on-resistance is recorded as *r*’. As long as the time constants of charging (*τ*_1_ = *r*’*C*) is far less than that of discharging (*τ*_2_ = *rC*), the capacitor *C* can be charged fast and discharge to resistor *r* slowly.

**Figure 10:**
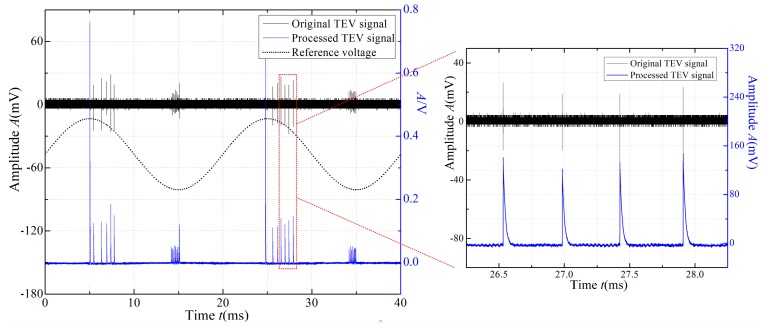
The original and processed TEV PD signals.

The original and processed TEV PD signals for A/D data collection are shown in Figure 10. The figure shows that the original TEV signal (time of duration: 0.6 μs), can be transferred to the low-frequency domain signal (time of duration: about 50 μs), but with a higher signal-to-noise ratio (SNR), which can be collected by the relatively low-speed acquisition unit. In our work, a low-power acquisition unit with 1 MS/s sampling rate, and A/D precision of 12 bits was selected to build the TEV sensing unit.

### 3.4. Structural Design of the Wireless TEV Sensors

**Figure 11:**
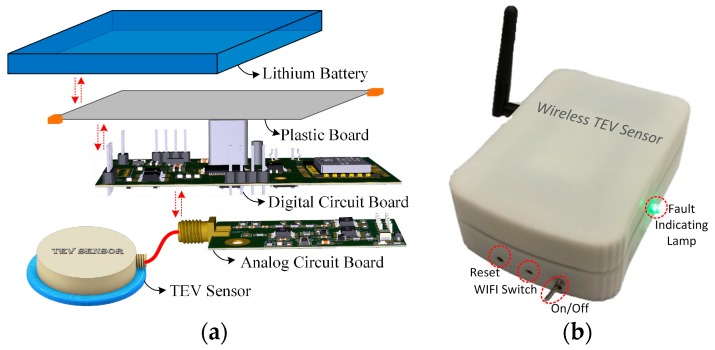
Wireless TEV sensor: (**a**) structural design diagram; and (**b**) physical picture.

Considering anti-interference, the wireless TEV sensors were rigidly designed with conductive material coating their outer surface. Each wireless TEV sensor was designed and manufactured, as shown in Figure 11, with dimensions 108 mm × 80 mm × 37 mm and weight 420 g. The power supply used with the signal acquisition and information transmission of the monitoring units were lithium batteries, thereby avoiding complex power line reconstruction. In order to improve the battery life, a combination of the periodic start mode and servo start mode was adopted in the monitoring strategy. That is, after the completion of a measurement task, the sensor unit enters deep sleep mode. In addition, device selection, hardware design, and power management used low-power mode. The output voltage and capacity of the lithium battery are 3.7 V and 4000 mAh, according to the theoretical calculation and evaluation, which guarantees normal work for at least two years when the self-starting period of the sensor is twice per day. In addition, the sensor is equipped with a fault indicating lamp—a tri-color LED with green, blue, and red lights representing the three alarm levels, normal, abnormal, and dangerous, respectively. A built-in permanent magnet on the bottom of the sensor enables it to be attached to metal surfaces in practical application.

### 3.5. Calibration of the Wireless TEV Sensors

**Figure 12:**
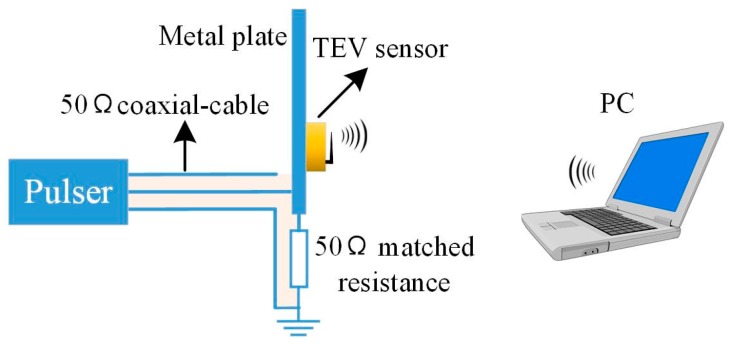
The experimental TEV PD calibration system.

In this study, a TEV PD calibration system, comprising a TEV signal source, signal transmission cable, matched resistance, wireless TEV sensor, and terminal computer, as shown in Figure 12, was designed based on the TEV signal generation and PD detection principle.

**Figure 13:**
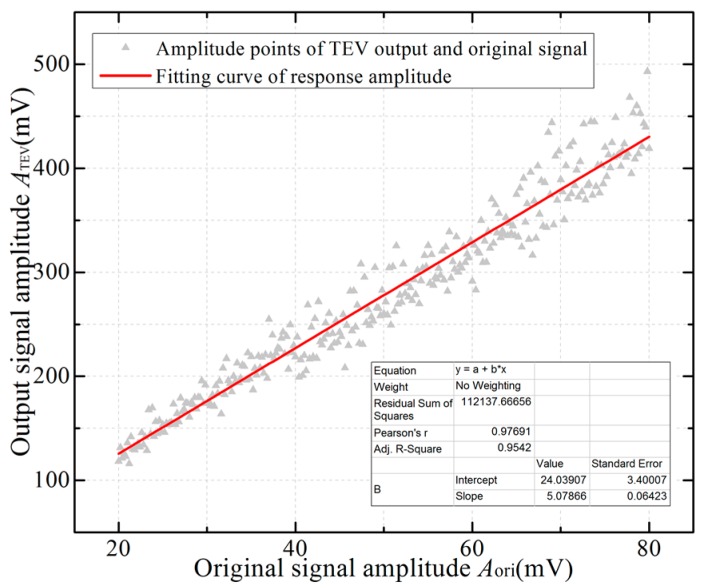
Amplitude calibration curve of wireless TEV sensor.

The TEV signal source is a pulse generator that outputs a periodic Gaussian pulse with risetime 11.7 ns (approximately 30 MHz) to imitate the TEV signal on the metal surface generated by EM waves. The metal plate is connected to a pulser via a 50 Ω coaxial-cable and grounded through a 50 Ω matched resistor. The TEV sensor measures the signal on the metal surface and sends the test result to the terminal PC via Wi-Fi. The pulse generator output amplitude ranges from 20 mV to 80 mV; the corresponding sensitivity and response characteristics of the sensor are shown in Figure 13.

As can be seen from Figure 13, the relationship between the TEV output and the original signal can be fitted using the following linear equation:

(18) $$A_{\mathrm{TEV}}=24.04+5.08\times A_{\mathrm{ori}}$$

where *A*_TEV_ and *A*_ori_ are the output amplitude (in mV) of the TEV sensor and the original signal, respectively.

Traditional PD detection with pulse current method [23] can measure the PD apparent charge of the HV electrical equipment and express discharge intensity in unit of pC, while TEV results are customarily expressed in logarithmic unit of dBmV to indicate the intensity of the discharge. The relation between the TEV signal (mV) and output result (dBmV) is as follows:

(19) $$\mathrm{dBmV}=20\log_{10}(\mathrm{mV})$$

According to Equations (18) and (19), the output result of a wireless TEV sensor satisfies the following equation:

(20) $$D=20\log_{10}(\frac{A_{\mathrm{TEV}}-24.04}{5.08})$$

where *D* is the logarithmic value of the sensor output with units in dBmV.

On the basis of the above analysis and research, the technical parameters and values of the wireless TEV sensor are shown in Table 2 and the relative error is estimated by the deviation between the measured value (*A*_TEV_) and the standard value (*A*_ori_). These sensors, whose technical parameters satisfy the requirement of detection application in the field, lay the foundation for establishing the WSN for medium-voltage (MV) switchgear monitoring.

## 4. WSN of TEV and Its Application Strategy

**Figure 14:**
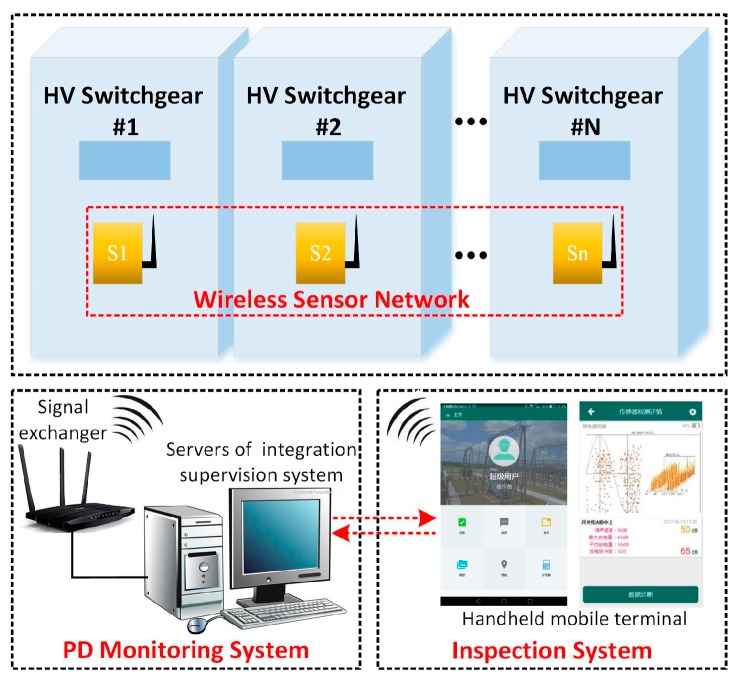
Application strategy of the wireless TEV sensor.

**Figure 15:**
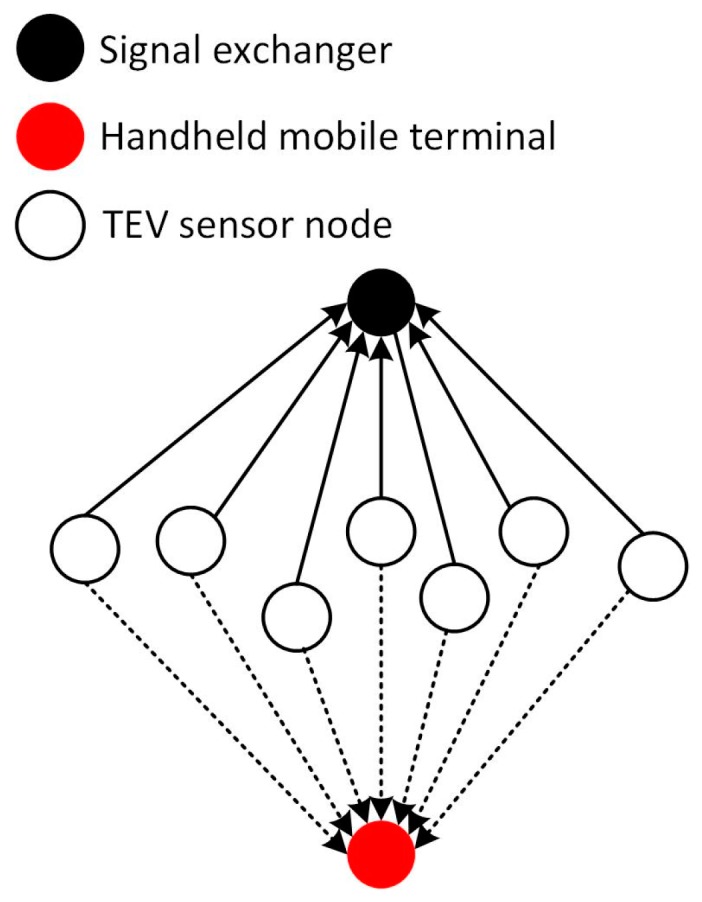
Topological graph of WSN: direct transmission model.

Based on the wireless distributed TEV sensor, an intelligent detection system and application strategy in the field, which combine the advantages of live testing and online testing, was developed. The construction of the TEV WSN and its corresponding topological graph is shown in Figure 14 and Figure 15, respectively. In general, the entire monitoring system consists of TEV WSN, signal exchanger, servers of the integration supervision system, and a handheld mobile terminal. In addition, the PD detection strategy includes two parts: the PD monitoring system and inspection system. For the PD monitoring system, when all of the MV/HV switchgear is operating normally, the sensing units regularly upload the characteristic data according to the preset monitoring density, for example, twice per day. When the test data are abnormal, the system increases the monitoring density, even changing into the real-time monitoring mode automatically, and sends the status information to the terminal server and operation and maintenance personnel. In terms of the inspection system, when it is time for the regular inspection of the HV switchgear or a switchgear failure warning occurs, the operation and maintenance personnel can carry the handheld mobile terminal to the switchgear room for further diagnosis onsite. Considering the complex EM interference onsite, a direct transmission model is adopted as the topological structure of the WSN to guarantee the stability and reliability of the entire system.

For the WSN, each sensing unit can operate independently according to the commands and the strategies set by the upper diagnostic layer and obtains the status of the switchgear with the warning level. To avoid uploading redundant data to the decision layer, onsite primary diagnosis results at a low warning level are kept in the sensing unit. If an uptrend is confirmed with historical data, the status data are uploaded to the diagnosis layer and the long-term real-time monitoring mode is activated. Before uploading the data and starting the real-time monitoring mode, a correlation analysis should be implemented on the short-term abnormal signal via Wi-Fi to estimate the background noise and recognize the real PD signals from the stochastic disturbance. The TEV PD signal coupled by one sensor unit is compared with the signals coupled by the adjacent units to exclude the external common interference. Further, the historical data, including PD magnitude and PD frequency, are used to determine whether an insulation fault status is developing or a sudden fault has occurred.

## 5. Switchgear PD Diagnostic Method Based on a Wireless Distributed TEV Sensing Unit

Most traditional PD detections rely on manual inspection and manual judgments. However, for the substations or power distributions that have a large number of switchgear, PD detections are time-consuming and labor intensive and, because of the empirical judgment required, misjudgment, which means that the normal state is mistakenly diagnosed as the failure state and missed judgment that means that the failure state is diagnosed as the normal state are unavoidable. Based on the sensing network, the cumbersome manual maintenance can be avoided, and the objectivity of data records can be improved. In addition, WSN can also realize front-end processing on PD signals, and adjust monitoring mode automatically according to the operation status of the power grid. In terms of the PD diagnostic method based on TEV WSN, the noise level of the entire sensing network is first evaluated. Then, according to the uploaded data, the elementary status diagnosis, which includes quantity analysis, trend analysis, and parallel analysis, is implemented to sift the abnormal switchgear and the corresponding danger degrees are determined. Finally, an in-depth status diagnosis is carried out to realize PD pattern recognition and the location of the switchgear.

### 5.1. In Situ Status Estimation

**Figure 16:**
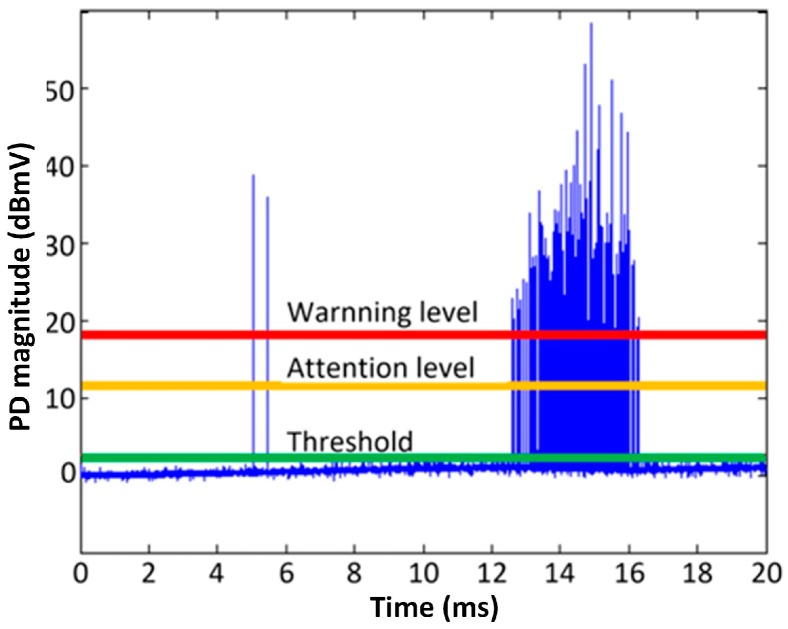
The three levels determined by the sensor unit.

In order to reduce data transmission and data redundancy, and increase the adaptability of the sensing network, the sensor unit needs to have primary diagnosis ability, including background noise estimation and historical trend analysis. Figure 16 shows an example of the three levels determined by the sensor unit in primary diagnosis—specifically, triggering threshold, attention level, and warning level—which is decided by the decision layer according to the empirical value, background noise level, and historical trend. The in situ status is recorded by the sensor unit for historical analysis. Only if the PD level exceeds the attention level are the full sampled data uploaded to the host PC.

### 5.2. Elementary Status Diagnosis

#### 5.2.1. Quantity Analysis

The risk levels—normal, attention, and warning—are determined comprehensively by empirical values, which derives from the statistical analysis of experimental data and failure cases. According to the TEV value in “dBmV”, the PD severity can be divided into the four ranges shown in Table 3. This offset analysis provides the basic information of a switchgear for in situ status estimation and correlation analysis without considering the interference and background noise level.

#### 5.2.2. Trend Analysis

Trend analysis is also applied in diagnosis based on the variation of the quantity with time. This occurs when the PD activity reaches an abnormal level; it is only applied to the intensive monitoring mode and is, therefore, not applicable during normal status. The time trend analysis is performed as follows.

The relative variation of quantity with time can be calculated using Equation (21):

(21) $$\Delta q\%=\frac{A_{t2}-A_{t1}}{A_{t1}\times \Delta t}\times 100\%$$

where *A_t_*_1_ and *A_t_*_2_ are the test quantity results before and after a time interval Δ*t*. Δ*t* is determined by the risk level obtained via quantity analysis, it can be 10 min, 1 h, 1 day, etc. According to the calculated variation, the insulation status is divided into two modes: developing insulation deterioration and sudden fault. If the variation exceeds 50%, the status is rated as an urgent risk level.

#### 5.2.3. Parallel Analysis

For the same type of switchgear operating under the same working condition, parallel analysis can be used to determine PD location and severity. The parallel analysis is performed as follows:

■ Calculate the average PD magnitude *A_av_* of both switchgear, including the target switchgear, at the same test time. Set the reference deviation value Δ*m_r_*%. Calculate the deviation of the target switchgear Δ*m*% using Equation (22):
  (22) $$\begin{array}{l} A_{av}=\frac{1}{k}\sum_{n=1}^{k}A\\ \Delta m\%=\frac{A-A_{av}}{A_{av}}\times 100\% \end{array}$$
■ If the Δ*m*% of the target switchgear is less than Δ*m_r_*%, the insulation status of the target switchgear is determined as “Normal”.
■ If the Δ*m*% exceeds Δ*m_r_*%, the insulation status of the target switchgear is determined as “Abnormal” and its risk level is estimated according to the criteria list in Table 4.

### 5.3. In-Depth Status Diagnosis—PD Pattern Recognition

#### 5.3.1. Acquisition of PRPD Diagram Based on Wireless TEV Sensor

As mentioned above, 1 MS/s sampling rate and A/D precision of 12 bits was adopted by the designed TEV sensing unit. Due to the internal storage limitation of the ARM chip, the sensor collects PD signals of only 20 ms each time, which is equal to one period of power frequency voltage (50 Hz) and there are 20,000 data points. To obtain the PRPD diagram, the terminal sends commands to the sensor and switches it into the PRPD collection mode, firstly. Then, the sensor collects the PD signal for the first time and extracts the peak value (*q*) and its corresponding coordinate (*k*, the sequence number among 20,000 points) of every PD pulse to build the array (*k_i_*, *q_i_*). After this array (*k_i_*, *q_i_*) has been updated from last collection, the internal clock of the ARM chip controls the triggering time of the next collection, which ensures that the time interval between the two collections is an integral multiple of 20 ms to strictly guarantee the phase consistency. When the collected PD number is sufficient or the collection time is long enough (these two parameters can be set by the terminal), the collection process is ended. The coordinate parameters (*k_i_*) can be converted to reference phase (*φ_i_*) by the following equation:

(23) $$\varphi_{i}=\frac{2\pi \times k_{i}}{20,000}$$

Eventually, the final data array (*φ_i_*, *q_i_*) is uploaded to the terminal for the display of PRPD diagram and further analysis.

**Figure 17:**
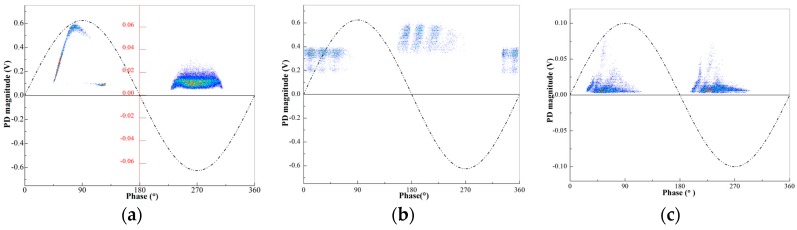
PRPD diagram of switchgear typical insulation defects: (**a**) needle-plate discharge caused by metal protrusion; (**b**) floating potential discharge caused by bad electrical contacts; and (**c**) surface discharge due to pollution of the insulator or metal particle on the insulator surface.

Measured by wireless TEV sensor, the PRPD diagrams of switchgear typical insulation defects are shown in Figure 17. Each PRPD diagram presents a distinct phase distribution feature.

#### 5.3.2. Feature Extraction of PD Image

**Figure 18:**
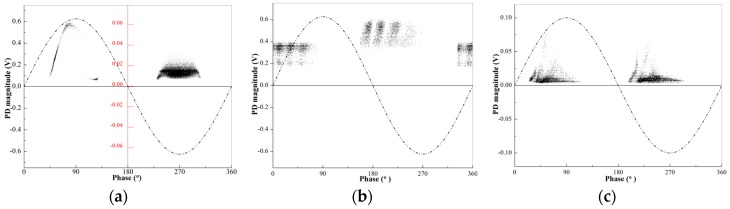
Corresponding grayscale images of PRPD diagram: (**a**) needle-plate discharge; (**b**) floating potential discharge; and (**c**) surface discharge.

Instead of the traditional PRPD features extraction, a grayscale image method is adopted to enhance the identification rate of the PD pattern in this diagnosis system. Grayscale images, which have the characteristics of discharge phase distribution, discharge amplitude, and discharge repetition rate, are one of the important and common characterization methods for PD fault identification. Typically, PD grayscale images can be obtained by calculating the grayscale value in the local area of PRPD diagram according to the following equation:

(24) $$h_{i,j}=255\times \frac{n_{i,j}}{n_{\max}}$$

where *h_i,j_* is the grayscale value of each pixel point; *n_i,j_* is the number of discharge; *n*_max_ is the maximum number of discharge and the grayscale value ranges from 0 to 255. The corresponding grayscale images of PRPD diagrams (Figure 17) for typical insulation defects of switchgear are shown in Figure 18.

Then, two-dimensional maximum margin criterion (2DMMC) [24] is used to reduce the dimensionality of the PD grayscale images by horizontal and vertical compression. A series of feature vectors are extracted from the converted matrix to construct the partial discharge image feature sets.

#### 5.3.3. PD Pattern Recognition Based on a Support Vector Machine

**Figure 19:**
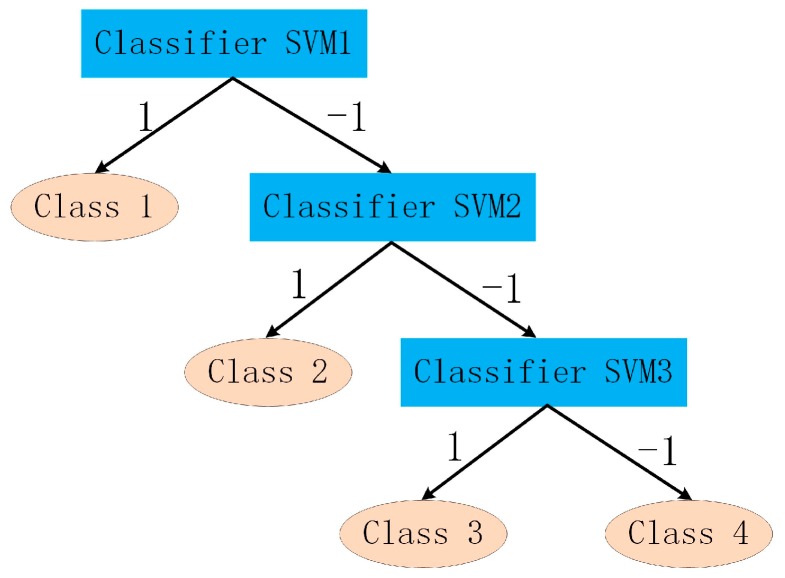
SVM classification model based on binary tree. Class 1: floating discharge; Class 2: surface discharge; Class 3: air-gap discharge; and Class 4: needle-plate discharge.

As a classification method based on structural risk minimization principle, a support vector machine (SVM) [25,26] is adopted to conduct PD pattern recognition of abnormal switchgear in this paper. Using non-linear mapping, the SVM maps input vectors to a high-dimensional feature space and approximates the objective function with optimal hyperplane. In addition, it has good generalization ability and can be used to solve the calculation of high-dimensional space well. For PD pattern recognition, the SVM needs to be extended to a multi-classifier [27]. Therefore, the construction of the SVM with a binary tree is designed to recognize four kinds of discharge patterns, as shown in Figure 19. The SVM classifier output values are ‘1’ and ‘−1’, respectively.

**Figure 20:**
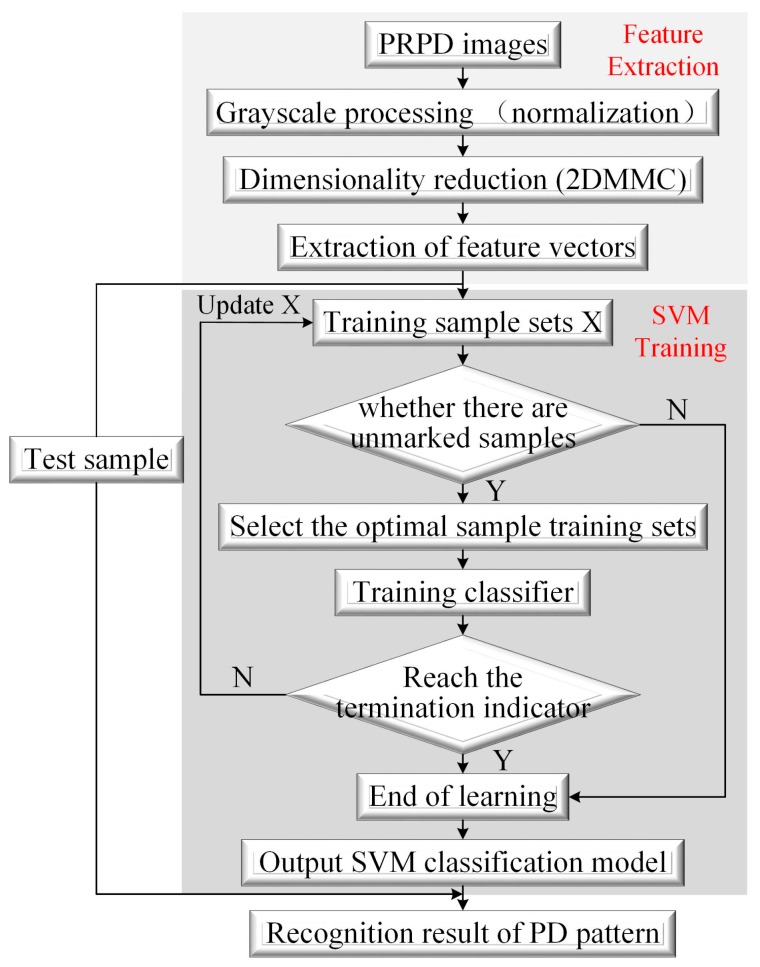
PD pattern recognition process based on an SVM.

The whole PD pattern recognition process is shown in the Figure 20. In general, its recognition rate is around 90% for four kinds of PD pattern, which satisfies the basic needs of onsite diagnosis.

### 5.4. Diagnosis Procedure

**Figure 21:**
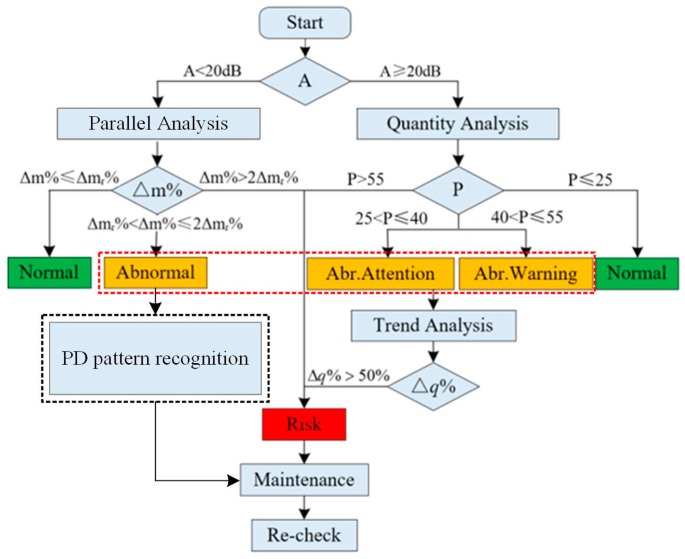
Diagnosis procedure in the decision layer.

On completion of the in situ status diagnosis in the sensing layer, the PD activity and the status information are uploaded into the diagnosis layer. Quantity analysis, trend analysis, parallel analysis, and PD pattern recognition are utilized in this process and a deterministic diagnosis result is provided for risk assessment. The detailed diagnosis procedure is shown in the Figure 21.

## 6. Conclusions

This paper proposed an innovative online PD detection system and corresponding application strategy based on a designed distributed TEV wireless sensor network, which can effectively promote the in-depth engineering application of TEV detection technology. The summary and main conclusions are as follows.

(1) The designed wireless TEV sensor can effectively couple PD signals with high response characteristics, high signal-to-noise ratios, low power consumption, good linearity, and anti-interference performance.
(2) With the advantages of online monitoring and inspection testing, the innovative self-adapting TEV WSN system has significant engineering application value for estimating the insulation condition of metal-enclosed switchgear.
(3) A two-step approach, consisting of elementary status diagnosis and in-depth status diagnosis, was developed to efficiently determine switchgear abnormality and its danger level.

## Figures and Tables

**Table 1:** Component values of TEV coupling module.

Component	*C*_1_	*C*_2_	*C*_3_	*R*_1_
Value	85 pF	1 pF	100 pF	5 kΩ

**Table 2:** Technical parameters of the wireless TEV sensor.

Index	Minimum Detectable Apparent Discharge Quantity	Bandwidth	Relative Error
Value	5 pC	1–30 MHz	≤5%

**Table 3:** Risk determination via quantity analysis.

Criterion, P (dB)	Risk Level	Description	Treatment
P ≤ 25	Normal	/	Routinization
25 < P ≤ 40	Abnormal	Attention	Shorten monitoring interval
40 < P ≤ 55		Warning	Shorten monitoring interval and upload results to host PD
P > 55	Dangerous	Urgent	Upload results to host PD and turn on real-time monitoring mode

**Table 4:** Risk determination via parallel analysis.

Criteria	Risk Level	Description	Treatment
Δ*m*% ≤ Δ*m_r_*%	Normal	/	Routinization
Δ*m_r_*% < Δ*m*% ≤ 1.5Δ*m_r_*%	Abnormal	Attention	Shorten monitoring interval
1.5Δ*m_r_*% < Δ*m*% ≤ 2Δ*m_r_*%		Warning	Shorten monitoring interval and upload results to host PD
Δ*m*% > 2Δ*m_r_*%	Dangerous	Urgent	Upload results to host PD and turn on real-time monitoring mode
